# Cognitive impairment in multiple sclerosis: diagnosis and monitoring

**DOI:** 10.1007/s10072-021-05165-7

**Published:** 2021-04-01

**Authors:** Virginia Meca-Lallana, Francisco Gascón-Giménez, Ricardo C. Ginestal-López, Yolanda Higueras, Nieves Téllez-Lara, Joan Carreres-Polo, Sara Eichau-Madueño, Jesús Romero-Imbroda, Ángela Vidal-Jordana, Francisco Pérez-Miralles

**Affiliations:** 1 Unidad de Enfermedades Desmielinizantes, Servicio de Neurología, Hospital Universitario de La Princesa, Madrid, Spain; 2 Unidad de Esclerosis Múltiple, Servicio de Neurología, Hospital Clínico Universitario, Valencia, Spain; 3 Servicio de Neurología, Hospital Clínico San Carlos, Madrid, Spain; 4 Instituto de Investigación Sanitaria del Gregorio Marañón, Hospital Gregorio Marañón, Madrid, Spain; 5 Servicio de Neurología, Hospital Clínico Universitario, Valladolid, Spain; 6 Servicio de Radiología, Hospital Universitari i Politècnic La Fe de Valencia, Valencia, Spain; 7 Servicio de Neurología, Hospital Universitario Virgen Macarena, Sevilla, Spain; 8 Servicio de Neurología, Hospital Regional Universitario de Málaga, Málaga, Spain; 9 Servicio de Neurología-Neuroinmunología, Centro de Esclerosis Múltiple de Cataluña (Cemcat), Hospital Universitario Vall d’Hebron, Barcelona, Spain; 10 Unitat de Neuroimmunología - CSUR Servicio de Neurología Hospital Universitari i Politècnic La Fe de Valencia, Valencia, Spain

**Keywords:** Multiple sclerosis, Cognitive dysfunction, Neuropsychological tests, Neurophysiological monitoring

## Abstract

**Introduction:**

Cognitive impairment (CI) has a prevalence of 45–70% in people with multiple sclerosis (MS), producing a negative impact on their quality of life, personal life, and work. Early detection of CI has become an important aspect to be considered for an adequate follow-up, to optimize social adaptation and to implement specific cognitive rehabilitation strategies. The aim of this work is to propose a suitable cognitive evaluation of patients with MS based on available and efficient tools for diagnosis and monitoring purposes well supported by literature review and clinical experience.

**Methods:**

A multidisciplinary panel of professionals from the field of neurology, neuropsychology, and neuroimaging performed a literature review of the topic of cognitive impairment assessment. This was combined and completed with their clinical experience to produce a set of recommendations.

**Results:**

Some limitations to cognitive evaluation are described: shortage of time and resources during the neurology consultation, scarceness or absence of specialized professionals’ availability, importance of tests adaptation, and doubts about its use to define therapeutic efficiency. We recommend a baseline and annual screening evaluation, and we suggest a baseline and periodic neuropsychological assessment. The latter ought to change to a recommendation with the presence of either positive screening test, or subjective to cognitive complaints, screening-test results and patient or family report mismatch, or in specific social/work situations.

**Conclusions:**

Cognitive evaluation should be performed on all patients diagnosed with MS and throughout follow-up. It is necessary to support the creation of multidisciplinary MS teams to optimize the evaluation and follow-up of MS patients.

## Introduction

Cognitive psychology defines several cognitive processes as the focus of neuropsychological evaluation, such as language, thinking, attention, memory, and emotions*.* Cognitive impairment (CI) is a common symptom in multiple sclerosis (MS) with a prevalence of 50–60%. It influences the patients’ quality of life, work, and social functioning [1–3]. The most frequently affected cognitive domains are information processing speed (IPS), complex attention, working memory, visuospatial ability, and executive functions [2, 4–8], with predominance of dysexecutive disorders in the progressive forms of MS and an amnestic profile in relapsing-remitting MS [9].

Cognition does not always correlate with the Expanded Disability Status Scale (EDSS) score. CI can be found in early MS [10, 11] and also in the asymptomatic forms of MS such as the Radiological Isolated Syndrome (RIS) [2, 3, 12–14]. The presence of CI at the time of diagnosis of MS is considered a poor evolution prognostic marker [11, 15]. The involvement of verbal memory and IPS, detected in early stages of MS, is predictive of more significant long-term disability. The early detection of CI is of utmost importance in order to ensure a correct social and work adaptation of the patient, and to implement specific cognitive rehabilitation strategies [16].

The Cognition Working Group is part of the EMDAT study group (Multiple Sclerosis Disease Activity Task Force) and is composed by neurologists and a neuropsychologist from different healthcare centers in Spain. The objective of this working group is twofold: to analyze the current difficulties to implement a systematic cognitive assessment (CA) in routine clinical practice and to provide a strategy to detect and monitor cognitive impairment in MS patients based on a systematic review of the literature and the clinical experience of the group.

## Cognitive impairment in multiple sclerosis

### Definition

Fischer and colleagues suggested that, based on the result of a neuropsychological evaluation, CI in MS can be defined by the presence of any of the following aspects [17]:

- A performance below 1.5 or 2 standard deviations (SD) compared to the normative mean in at least 20–30% of the test parameters.
- A performance below 1.5 or 2 SD in at least two cognitive domains.

It has been considered that these two definitions of CI offer more reliable results [18].

### Vulnerability

The presence and evolution of CI is very heterogeneous among individuals with MS. There is a relationship between magnetic resonance imaging (MRI) data and cognition in MS. However, CI can be only partly explained by the presence of MRI lesions [19], or by other factors such as cognitive reserve (CR) that could explain the discrepancy between MRI and CI. The CR describes the ability to adapt cognitive activity despite brain damage [20] and can be measured with specific test [21] such as the Cognitive Reserve Index Questionnaire (CRIq). Vulnerability of developing CI in MS patients is higher when they present with high lesion load and cerebral atrophy at baseline MRI combined with low scores in CR test and cognitive tests at the beginning of the disease [22].

In order to detect CI earlier and improve prognosis, it has been suggested that the following three aspects should be considered: to perform early and periodic cognitive evaluations, to offer neuropsychological interventionist programs, and to consider the presence of CI as another possible poor prognostic factor when selecting disease-modifying treatments [16, 23].

## Importance and utility of cognitive function measurement in multiple sclerosis

CI has an impact on different aspects of MS patients’ daily life functioning [22]. The periodical evaluation of the cognitive function in MS would provide [23]:

- Knowledge of the baseline condition of the patient.
- Information about any cognitive change throughout the disease, either related with disease progression or cognitive relapses [24].
- Prognostic information in order to select a specific disease modifying treatment, as CI is considered a poor prognostic factor with a possible negative impact on treatment adherence.
- Information for the patient and family or caregivers about the presence of the CI that might help them to resolve doubts, and facilitate social work and socio-family adaptation.
- A working scenario to plan an early therapeutic intervention (cognitive rehabilitation and enhancement of the cognitive reserve).

### Therapeutic approach to cognitive impairment

Previous studies have shown that neuropsychological interventions produce positive effects on cognitive performance and other associated abilities, but results from clinical trials are still inconclusive due to methodological limitations [25]. Neuropsychological intervention aims to implement strategies to compensate for or recover cognitive deficits, to promote awareness of CI by the patients and their environment, and to work on the impact that CI has on their daily life activities.

A Cochrane review of 20 studies about the effects of cognitive rehabilitation concluded that there was a low level of evidence for its recommendation; however, the comparison of the different rehabilitation strategies was difficult because of the heterogeneity of interventions and outcome measures [25]. It was also reported that some studies showed that cognitive training improved immediate and working memory abilities and the use of specific rehabilitation training programs did have a positive impact on attention and immediate and verbal memory [25]. In a later review, the results of cognitive intervention on executive function and attention (frequently impaired in MS patients) were more consistent [26].

Functional neuroimaging facilitates the establishment of a correlation between image and cognitive abilities in MS [27] and has been used to study the efficacy of cognitive rehabilitation programs [28]. New research explores the concept of adaptive versus maladaptive neuroplasticity in relation with cognitive rehabilitation programs. Chiaravalloti and colleagues observed that there is an activity increase locally in tissue immediately surrounding a demyelinating lesion, which appears early in MS patients even without CI. This was often associated with intact cognitive functioning and was interpreted as adaptive neuroplasticity. Extra-region activation is considered adaptive inefficiency and is associated with worse cognitive performance [29].

## Current status of cognitive assessment: usual difficulties

We considered that the main limiting factors for CA in patients with MS are the following:

- Time limitation during the neurologist consultation.
- Lack of material and human resources, especially trained personnel and special equipment [6, 12, 30].
- Limited availability of neuropsychologists with specific training in MS working in MS centers.
- Lack of validated tests and normative data that account for language differences ​and different educational levels to obtain reliable results [2, 6].
- Limited evidence about therapeutic interventions, since research into the effect of pharmacological treatments on the cognitive status of patients with MS has shown inconsistent results [31].

## Tools to measure cognitive impairment in multiple sclerosis

Neuropsychological tests and questionnaires are tools that allow professionals to analyze and quantify cognitive abilities individually and to compare individuals’ performances. Adequate training for administration and interpretation of the neuropsychological testing is mandatory. In MS, some tools have demonstrated a higher sensitivity in the detection of CI and they are the ones used more often. As CI in MS shows a distinctive profile compared to other neurodegenerative diseases, the neuropsychological tools for CA must be specific (Table 1) [32–42].

**Table 1:** Recommended tests for the evaluation of specific cognitive functions (based on Arnett and Forn, 2007) [32]

Cognitive domain	Test
Orientation	Orientation subtest WMS-III [33]
Processing speed	PASAT [34, 35]
Sustained attention	CPT [36]SDMT [34]
Memory	
Immediate verbal memory	Direct Digit Subtest of WMS-III [33] or WAIS- III [37]
Verbal working memory	Inverse Digit Subtest of WMS-III [33] or WAIS- III [37] Letter Number Sequencing subtest of WAIS-III [37]
Auditory verbal learning and long-term memory/recall	Spain/Complutense Verbal learning Test (TAVEC) [38]
Executive functioning	
Visuospatial learning and visuospatial long-term memory	10/36 SPART [34, 35]
Phonetic verbal fluency	F, A, S [39]
Semantic verbal fluency	Animals, fruits, vegetables [30]
Planification	Tower of London [40]
Abstract reasoning	WCST [41]
Abstract verbal reasoning	Similarities subtest of WAIS-III [37]
Visuospatial functions	JLOB [42]

WMS-III: Wechsler Memory Scale III; PASAT: Paced Auditory Serial Addition Test; CPT: Continuous Performance Test; SDMT: Symbol Digit Modalities Test; WAIS-III: Wechsler Adult Intelligence Scale III; 10/36 SPART, 10/36 Spatial Recall Test; WCST: Wisconsin Card Sorting Test; JLO: Judgement of Line Orientation

CA relies on both quantitative and qualitative information [43] and the neuropsychological assessment can be performed using either a screening test or a cognitive-specific neuropsychological tool.

### Screening tests for cognitive assessment

Cognitive screening is performed when cognitive status is previously unknown [44]. These tests are brief, simple and with high sensitivity and specificity [45]. The most utilized in MS are the following (see Table 2) [46–49]:

- MSNQ (Multiple Sclerosis Neuropsychological Questionnaire)

**Table 2:** Rapid screening tests to detect cognitive impairment in patients withmultiple sclerosis

	Test		
Domain	PASAT [46, 47]	SDMT [48]	MSNQ^a^ [49]
Auditory processing speed and working memory	+		
Visual processing speed and working memory		+	
Information processing speed			+
Verbal learning			+
Mean score (standard deviation)	46.7 (9.1)^b^50.4 (9.7)^c^	Depends on age and years of education	Not applicable
Duration	10-15 minutes	90 seconds(5 minutes including instructions)	Self-administered

MSNQ: Multiple Sclerosis Neuropsychological Questionnaire; PASAT: Paced Auditory Serial Addition Test; SDMT: Symbol Digit Modalities Test

^a^ MSNQ-Informant report correlates with Boston Naming Test (-0.45, p<0.001), CVLT-II Total Recall Trials 1-5 (-0.53, p<0.01), CVLT-II Delayed Recall (-0.43, p<0.001), BVMT-R Delayed Recall (-0.43, p<0.001), Trail Making Test (0.55, p<0.01), Paced Auditory Serial Addition (-0.47, p<0.001), WCST Perseveration Responses (0.37, p<0.01)

^b^ Scores up to 12 years of education and ^c^ scores for 12 or more years of education, according to Rao et al (1991) [7]

The MSNQ is a questionnaire with 15 items that evaluate the neuropsychological competences in daily life activities, by assessing different cognitive domains (attention, memory, processing speed, etc. [30]). There are two versions of the test: one version for the patient (MSNQ-self report or MSNQ-S) and another one for the caregiver (MSNQ-informant report or MSNQ-I). The usefulness of MSNQ-S is limited since it is highly depended on depression and mood disorders [30]. A cut-off score of ≤24 in MSNQ-S will correctly classify 68% of the patients, with a sensitivity of 0.83 and a specificity of 0.6; a score >22 at MSNQ-I will correctly classify 85% of the patients with a sensitivity of 0.87 and a specificity of 0.84 [49, 50].

The MSNQ-I is useful to identify risk groups. It is not influenced by the patient’s depression, and correlates well with working memory, learning, and executive and visuospatial functions [50]. It also correlates with certain MRI parameters (lesion volume and brain atrophy), secondary progressive course, and work disability. It has been shown that an increment of one point in the MSNQ-I increases the risk of CI by 6.5% [30]. The main advantage of this test is that it is easy, short, and easily reproducible [12, 30, 51]. The main drawbacks are the uncertainty of the informant’s depression effect on the score, the poor CI detection capability when patients show low repercussion effects on their daily lives, and the lack of the MSNQ-I score for unaccompanied patients [12, 30].

- PASAT 3” (3-s Paced Auditory Serial Addition Task)

The PASAT 3” is used as a multi-domain measure, informing about complex attention processes, working memory, and executive functions. Single digits are presented every 3 s and the patient must add each new digit to the one immediately prior to it. It was included in the Multiple Sclerosis Functional Composite (MSFC) as a measure of disease monitoring and to measure cognition in clinical trials [52].

Is a short test (10–15 min) with good sensitivity to detect CI in MS patients, offering cognitive information independently of the speed of processing [53]. It can distinguish between healthy controls and MS patients with a cut-off score of 43 (sensitivity of 0.82 and a specificity of 0.69) and CI in MS patients with a cut-off score of 40.1 (sensitivity 0.74 and specificity 0.65) [54]. Nevertheless, still there is not a general cut-off point to define a clinically significant progression [55], it can be stressful for the patient, usually requires specific material and its outcome depends on different factors, such as education or age. Those are the reasons why it has been replaced in many clinical trials by the SDMT (Symbol Digit Modality Test) [51].

- SDMT (Symbol Digit Modality Test)

In this test, the patients are shown a visual key that matches numbers and symbols on the top of a sheet. Then, they must specify the correct number for each of the symbols presented as fast and as accurately as they can during 90 s [51]. This test assesses processing speed and visual working memory. The SDMT is fast, easy, and has a low cost [2, 3]. It is sensitive for the detection and change of CI in MS [6]. The score of the test correlates with CI, MRI (lesion load, ventricular volume, cortical, and deep grey matter atrophy), and with the patient’s functional status [2, 6]. The SDMT has a higher capacity in distinguishing patients with MS from controls. Of the cognitive tests included in the BRB-N (Rao Brief Repeatable Neuropsychological Battery) and the MACFIMS (Minimal Assessment of Cognitive Function in Multiple Sclerosis), the SDMT is the best test for the detection of CI in MS [6, 12, 35, 56]. An optimal cut-off score of 55 hits has been suggested, correctly categorizing 72% of patients, with a sensitivity of 0.82, specificity of 0.60, and a positive predictive value (PPV) of 0.71 [49, 50]. A decrement of 4 or more points or 10% of the SDMT or 0.5 SD could be considered a clinically significant measure of cognitive worsening [57]

This is a fast test (5 min), easily reproducible, and does not require specific neuropsychological training for its administration [2, 51]. Compared to the other tests, it is more sensitive [57], requires less time, does not require any electronic equipment, and has a prognostic value correlating with the disability degree at 5 and 7 years [2, 13, 51]. As a drawback, it is just a screening test that does not offer any information about other cognitive domains.

### Neuropsychological evaluation batteries

A more extensive neuropsychological evaluation allows for a more detailed study of several cognitive domains. The use of neuropsychological batteries is recommended after either a positive screening test, subjective cognitive complaints reported by the patient or his caregiver, discordance between clinical perception/screening tests or in specific socio-labor situations [16, 18]. The contents of the three most used neuropsychological batteries in MS are detailed in Table 3 [6, 58, 59].

**Table 3:** Neuropsychological batteries for the evaluation of patients withmultiple sclerosis

	Batteries		
Domain	MACFIMS [58]	BRNB [59]	BICAMS [6]
Auditory processing speed and working memory	PASAT	PASAT	
Visual processing speed and working memory	SDMT	SDMT	SDMT
Verbal/auditory memory	CVLT2	SRT	CVLT2
Visual/spatial memory	BVMTR	10/36 SPART	BVMTR
Language	COWAT	COWAT	
Spatial processing	JLO		
Executive functioning	DKEFS Sorting		
Duration	90 minutes	50 minutes	20 minutes

CVLT2: California Verbal Learning Test second edition; SRT: Selective Reminding Test; BVMTR: Brief Visuospatial Memory Test Revised; 10/36 SPART: 10/36 Spatial Recall Test; COWAT: Controlled Oral Word Association Test; JLO: Judgement of Line Orientation; DKEFS: Delis-Kaplan Executive Function System

#### Brief International Cognitive Assessment For Multiple Sclerosis (BICAMS)

It is a brief battery to be used as screening of cognitive status that can be administered by health personnel without specific training in about 15 min. It evaluates processing information speed with the SDMT, verbal memory with the CVLT-II (California Verbal Learning Test), and visual memory based on the result of the BVMT-R (Brief Visuospatial Memory Test revised), excluding scales of executive and visuospatial functions. If the assessment time is limited, the authors recommend to shorten the BICAMS and administer only the SDMT component.

CVLT-II evaluates learning and memory of verbal material. From all the indexes that this test offers, the BICAMS authors suggest the use of only the first five learning trials because this reduces time and possible effects of fatigue. This way of execution has been validated with MRI and brain atrophy measurements [58, 60, 61].

The BVMT-R assesses visual learning and memory. The protocol suggested in the BICAMS includes only the first three recall trials given their significant relationship with MRI parameters, brain atrophy [58, 60, 61], and functioning of diencephalic nuclei [60, 62]. It has shown a greater discriminative validity and sensitivity for visuospatial memory, with the advantage that it does not require specialized material and has no ceiling effect, although the functionality of the upper limbs may influence its outcomes [12, 56].

#### Brief Repeatable Battery-Neuropsychology (BRB-N)

This battery is composed by the SDMT, the PASAT 3”, the Selective Reminder Test (SRT), the 10/36 Spatial Recall Task (SPART), and the Word List Generation (WLG). The SRT is a verbal learning and memory test, the SPART assesses visual learning and memory, and the WLG assesses verbal fluency. In theory, the BRB-N is administered in 30–35 min although, depending on the patient’s capacity, it can take longer. Its administration and interpretation require a professional with training in neuropsychology. It has high sensitivity (67–71%) and specificity (85–94%). The BRB-N has an alternative version to avoid learning bias [34] and is available in Spanish [35]. As a disadvantage, some cognitive functions are poorly evaluated, or excluded, increasing the risk of false negatives.

#### Minimal Assessment of Cognitive Function in Multiple Sclerosis (MACFIMS)

It has been considered an improved version of the BRB-N [56] as it evaluates all cognitive functions affected in MS. In the MACFIMS visuospatial function tasks such as the Benton Line Orientation Judgment (JOLB)*,* the PASAT paced every 2 s and the Denis-Kapplan Battery (D-KEFS) Chart Classification trial for executive functioning were added.

Modifications to verbal memory measures are introduced, including the California Learning Verbal Test III, which replaced the SRT; the measurement of visual memory is changed to the Brief Visuospatial Memory Test-Revised (BVMT-R); and the measure of language fluency to the COWAT. It is administered in about 90 min. Adequate training in clinical neuropsychology is required [58].

## Recommendations and suggestions from the EMDAT group

Different factors should be considered when performing the CA, such as CR, emotional status (anxiety and/or depression), fatigue, and concomitant pharmacotherapy [16].

### When and where to perform cognitive assessment

CA should be performed in a quiet place without distractions. It is not recommendable to perform the assessment during the following month after a relapse or corticosteroid treatment, as they have a detrimental effect on memory [6]. It is convenient to conduct the assessments at the same time of the day to avoid possible biases of fatigue during the follow-up. Initially, it should be performed annually or biannually, and then, according to clinical criteria. It is necessary to take into account that certain medications may influence cognitive performance and also the patient mood [16, 18]. It is recommended that the baseline evaluation is performed as soon as possible after the diagnosis of MS, with subsequent periodic follow-ups.

### Neuropsychological evaluation according to available recourses in different healthcare centers (Fig. 1)

Availability of time and human resources varies between different hospitals, which may interfere with a correct CA. It is suggested that all patients should have a baseline and annual screening test completed, and a baseline and periodic neuropsychological evaluation should be performed on all patients at a suggestion level. Recommendation for follow-up evaluations should be made for any of the following scenarios: (i) a positive screening test; (ii) a negative screening result but with a subjective cognitive complaint reported either by the patient or family members; (iii) worsening of cognitive impairment that will require a complete evaluation; (iv) any social or working negative impact.

**Fig. 1:**
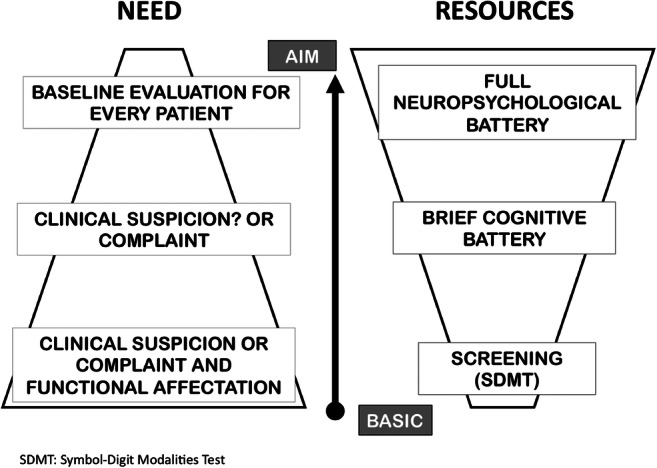
Description of current reality considering factors of need and available resources

### Centers with an available neuropsychologist in their staff

We recommend the BRB-N battery, and the addition of specific questionnaires to measure mood (BDI-II or HADS), fatigue (MSFIS), and quality of life (MSQoL-54) (See Table 4).

**Table 4:** Description of different cognitive scales used in MS (based on Sumowskyet al 2018) [22]

Test	Type of test	Cognitive domain assessed	Advantages	Disadvantages	Recommendations
SYMBOL DIGIT MODALITY TEST (SDMT)	Screening	IPSVisual work memory	Fast (90’’)Sensitive testWell toleratedAdditional versions available for follow-up testingNo learning or language bias	Results can be influenced by other cognitive functionsDoes not assess other cognitive domains	Useful for screening and follow-upUseful in clinical trialsComputerizable
PACED AUDITORY SERIAL ADDITION TASK (PASAT 3”)	Screening	IPSComplex attention processesWorking memoryExecutive function	Fast (5-10’)Applied by auditory stimuliModerate sensitivityIt is a well-known test in MS	Ceiling effectEducation level, stress and practice effects can influence results.It may generate frustration on the patient	Not recommended for clinical trialsUseful in screening and follow-upUseful if there is any visual or motor impairmentStandardize a way to quantify and interpret qualitative measures
SELECTIVE REMINGINGTEST (SRT)	Part of a battery	Verbal memory	High sensitivity(Unvalidated) versions available for follow-upIt affects the recovery of long-term memory instead of short-term recall	Normative data is missingOnly measure selective remindingIt neither measures ejective functions nor other cognitive domains	Useful in clinical trials for a follow-up evaluation of verbal memoryIt should be administered by a neuropsychologist
CALIFORNIA LEARNING VERBAL TEST(CVLT-II)	Part of a battery	Verbal memory	High sensitivityAvailable in Spanish (TAVEC)Good test-retest reliability	Only one retest versionIt needs 20’ for the long-delay recall assessmentNot computerizable	It should be administered by a neuropsychologistUseful to assesses verbal memory in clinical trials
BRIEF VISUAL MEMORY TEST REVISED (BVMT-R)	Part of a battery	Visual-spatial processing	High sensitivityFast to be administeredRetest versions availableWell toleratedIncludes recognition task	High inter-rater variabilityIt has no normative data in SpainRequires normal motor skills	Useful in assessment of visual memoryCan be used in trials.It should be administered by a neuropsychologist
SPATIAL RECALL TEST(10/36 SPART)	Part of a battery	Visual Memory	It doesn’t require normal motor skillsFree of semantic contentEasy to rate	Low sensitivityNo normative dataNo recognition task	Not as monitoring measure in Clinical trials.More useful than BVMT-R if motor skills are impairedIt should be administered by a neuropsychologist
CONTROL ORAL WORD ASSOCIATIONTEST(COWAT)	Part of a battery	Verbal fluencyExecutive function	Fast (3’)Moderate sensitivityWell tolerated	Can be influenced by education level and language bias.Results correlated with IPS and executive functionOnly phonetic fluencyErrors and strategies not analysed	Not in clinical trials or clinical monitoringIt should be administered by a neuropsychologistStandardize a way to quantify and interpret qualitative measures (executive function)
BENTON JUDGMENT OF LINE ORIENTATION(JLO)	Part of a battery	Visuospatial skills	Fast (3’)ReliableWell tolerated	Low sensitivity	Not in clinical trials or clinical monitoringIt should be administered by a neuropsychologistStandardize a way to quantify and interpret qualitative measures (executive function)
STROOP TEST	Independent Test	Inhibitory controlExecutive function	Fast (90”x3)Offers a measure of IPS (reading test) and interference resistanceIt is a verbal test; it does not require fine motor skills.	Requires normal visionIPS can influence the results.	Not in clinical trials or clinical monitoringIt should be administered by a neuropsychologist
MS QUALITY OF LIFE(MSQoL-54)	Self-administered questionnaire	Quality of life	Validated in SpanishMental and physical scalesIt includes functional, emotional, sexual and cognitive aspects.	Without normative dataIt is a long testQuestionable sensitivity for retesting	Measurement of impact on quality of lifeOutcome in clinical trials and monitoring
MODIFIED FATIGUE IMPACT SCALE (MFIS)	Self-administered questionnaire	Impact of fatigue	Distinguishes fatigue related to MSShort versionReliableIncludes physical, cognitive and social subscales	Without normative scales in Spanish	Part of evaluation batteryNot in clinical trials or clinical monitoring

BRB: Brief Repeatable Battery of Neuropsychological Tests; IPS: Information processing speed; MACFIMS: Minimal Assessment of Cognitive Function in Multiple Sclerosis

### Centers with limited human resources

If there is limited access or no availability of a neuropsychologist or neurologist with specific training, or there is limited time for each patient, the SDMT test is recommended as cognitive screening. If health caregivers with specific training are available, it is suggested to also administer the MSQN. A complete neuropsychological evaluation is recommended with abnormal SDMT scores [40–43] or when this score diminishes a 10% of the normal value or a score of 4 points below previous SDMT values. The cognitive screening test should neither replace a neuropsychological assessment nor be used for diagnosis since this might produce an increase of false negatives [3, 12].

### Evolution and follow-up

In case of normal results in cognitive screening tests or in cases with mild CI, no repercussion on daily life activities, and not classifiable as dementia [63], an annual evaluation is recommended for the first 2 years of follow-up, and thereafter depending on clinical evolution. In case of moderate or severe cognitive impairment, annual or biannual evaluations are recommended. After that they should be spaced out according to clinical criteria, until the patient meets the criteria for dementia, in which case stopping them is suggested. Intermediate evaluations are recommended if cognitive changes are noted.

## Next challenges

Multiple tools for a complete CA require specialized training in cognition, and they need to be carried out by a multidisciplinary team. The incorporation of neuropsychologists in Neurology Services and specifically in MS Units would improve patient care. It is necessary to standardize cognitive tests used in MS to homogenously quantify cognitive impairment. Causes of inter-patient cognitive variability are unknown [22]; therefore, analysis of cases with brain damage documented by MRI but normal neuropsychological results become important. It has been observed that MS patients with a high educational level in the initial phases of the disease have similar neuropsychological results compared to healthy controls the only exception being the processing speed [13]. This fact could suggest that compensating mechanisms are less efficient in protecting against deterioration of IPS; therefore, it would be essential to investigate how to empower them. There are critical radiological biomarkers such as brain atrophy related to CI [3], but they are not generally available in clinical practice. It has been described that the anti-inflammatory effect of some immunomodulation drugs could be beneficial in cognition [64]. However, there is no evidence that patients with CI benefit from a specific disease-modifying treatment, or from a change in the treatment. Finally, given that MS is a very complex disease, it would be ideal to have objective risk markers to identify patients with MS at risk of developing CI before presenting it.

## Conclusions

Given that CI in MS is frequent and represents a prognostic factor for disability in the long term, a complete CA must be performed on all patients at the time of diagnosis. CA allows informing and advising patients to facilitate their daily life, as well as to promote and design rehabilitation strategies for cognitive stimulation. It is necessary to include specialized professionals in the multidisciplinary EM teams to optimize the evolution and follow-up of patients.

## References

[1] Rao SM, Leo GJ, Ellington L, Nauertz T, Bernardin L, Unverzagt F (1991). Cognitive dysfunction in multiple sclerosis. II. Impact on employment and social functioning. Neurology.

[2] Van Schependom J, D’hooghe MB, Cleynhens K (2014). The Symbol Digit Modalities Test as sentinel test for cognitive impairment in multiple sclerosis. Eur J Neurol.

[3] Deloire MSA, Bonnet MC, Salort E, Arimone Y, Boudineau M, Petry KG, Brochet B (2006). How to detect cognitive dysfunction at early stages of multiple sclerosis?. Mult Scler.

[4] Labiano-Fontcuberta A, Mitchell AJ, Moreno-García S, Benito-León J (2014). Cognitive impairment in patients with multiple sclerosis predicts worse caregiver’s health-related quality of life. Mult Scler J.

[5] Chiaravalloti ND, DeLuca J (2008). Cognitive impairment in multiple sclerosis. Lancet Neurol.

[6] Langdon DW, Amato MP, Boringa J, Brochet B, Foley F, Fredrikson S, Hämäläinen P, Hartung HP, Krupp L, Penner IK, Reder AT, Benedict RHB (2012). Recommendations for a Brief International Cognitive Assessment for Multiple Sclerosis (BICAMS). Mult Scler J.

[7] Rao SM, Leo GJ, Bernardin L, Unverzagt F (1991). Cognitive dysfunction in multiple sclerosis. I. Frequency, patterns, and prediction. Neurology.

[8] Rocca MA, Amato MP, De Stefano N et al. MAGNIMS Study Group (2015). Clinical and imaging assessment of cognitive dysfunction in multiple sclerosis. Lancet Neurol 14(3):302-317 10.1016/S1474-4422(14)70250-9 25662900

[9] Zakzanis KK (2000). Distinct neurocognitive profiles in multiple sclerosis subtypes. Arch Clin Neuropsychol.

[10] Feinstein A, Kartsounis LD, Miller DH, Youl BD, Ron MA (1992). Clinically isolated lesions of the type seen in multiple sclerosis: a cognitive, psychiatric, and MRI follow up study. J Neurol Neurosurg Psychiatry.

[11] Glanz BI, Holland CM, Gauthier SA (2006). Cognitive dysfunction in patients with clinically isolated syndromes or newly diagnosed multiple sclerosis. Mult Scler J.

[12] Benedict RHB, Zivadinov R (2006). Predicting neuropsychological abnormalities in multiple sclerosis. J Neurol Sci.

[13] Deloire M, Ruet A, Hamel D, Bonnet M, Brochet B (2010). Early cognitive impairment in multiple sclerosis predicts disability outcome several years later. Mult Scler.

[14] Labiano-Fontcuberta A, Martínez-Ginés ML, Aladro Y, Ayuso L, Mitchell AJ, Puertas-Martín V, Cerezo M, Higueras Y, Benito-León J (2016). A comparison study of cognitive deficits in radiologically and clinically isolated syndromes. Mult Scler J.

[15] Zipoli V, Goretti B, Hakiki B, Siracusa G, Sorbi S, Portaccio E, Amato MP (2010). Cognitive impairment predicts conversion to multiple sclerosis in clinically isolated syndromes. Mult Scler J.

[16] Kalb R, Beier M, Benedict RHB, Charvet L, Costello K, Feinstein A, Gingold J, Goverover Y, Halper J, Harris C, Kostich L, Krupp L, Lathi E, LaRocca N, Thrower B, DeLuca J (2018). Recommendations for cognitive screening and management in multiple sclerosis care. Mult Scler J.

[17] Fischer M, Kunkel A, Bublak P, Faiss JH, Hoffmann F, Sailer M, Schwab M, Zettl UK, Köhler W (2014). How reliable is the classification of cognitive impairment across different criteria in early and late stages of multiple sclerosis?. J neurol sci.

[18] Amato MP, Morra VB, Falautano M, Ghezzi A, Goretti B, Patti F, Riccardi A, Mattioli F (2018). Cognitive assessment in multiple sclerosis-an Italian consensus. Neurol Sci.

[19] Benedict RH, Zivadinov R (2011). Risk factors for and management of cognitive dysfunction in multiple sclerosis. Nat Rev Neurol.

[20] Stern Y (2002). What is cognitive reserve? Theory and research application of the reserve concept. J Int Neuropsychol Soc.

[21] Nucci M, Mapelli D, Mondini S (2012). Cognitive Reserve Index questionnaire (CRIq): a new instrument for measuring cognitive reserve. Aging Clin Exp Res.

[22] Sumowski JF, Leavitt VM, Rocca MA, Inglese M, Riccitelli G, Buyukturkoglu K, Meani A, Filippi M (2018). Mesial temporal lobe and subcortical grey matter volumes differentially predict memory across stages of multiple sclerosis. Mult Scler J.

[23] Cheng EM, Crandall CJ, Bever CT, Giesser B, Haselkorn JK, Hays RD, Shekelle P, Vickrey BG (2010). Quality indicators for multiple sclerosis. Mult Scler J.

[24] Pardini M, Uccelli A, Grafman J, Yaldizli O, Mancardi G, Roccatagliata L (2014). Isolated cognitive relapses in multiple sclerosis. J Neurol Neurosurg Psychiatry.

[25] Rosti-Otajärvi EM, Hämäläinen PI (2014). Neuropsychological rehabilitation for multiple sclerosis. Cochrane Database Syst Rev.

[26] Mitolo M, Venneri A, Wilkinson ID, Sharrack B (2015). Cognitive rehabilitation in multiple sclerosis: a systematic review. J Neurol Sci.

[27] DeLuca GC, Yates RL, Beale H, Morrow SA (2015). Cognitive impairment in multiple sclerosis: clinical, radiologic and pathologic insights. Brain Pathol.

[28] Tomassini V, Matthews PM, Thompson AJ (2012). Neuroplasticity and functional recovery in multiple sclerosis. Nat Rev Neurol.

[29] Chiaravalloti ND, Genova HM, DeLuca J (2015). Cognitive rehabilitation in multiple sclerosis: the role of plasticity. Front Neurol.

[30] O’Brien A, Gaudino-Goering E, Shawaryn M (2007). Relationship of the Multiple Sclerosis Neuropsychological Questionnaire (MSNQ) to functional, emotional, and neuropsychological outcomes. Arch Clin Neuropsychol.

[31] Miller E, Morel A, Redlicka J, Miller I, Saluk J (2018). Pharmacological and non-pharmacological therapies of cognitive impairment in multiple sclerosis. Curr Neuropharmacol.

[32] Arnett P, Forn C (2007). Neuropsychological evaluation in multiple sclerosis. Rev Neurol.

[33] Wechsler D (2004). Wechsler Memory Scale III.

[34] Boringa JB, Lazeron RHC, Reuling JEW (2001). The brief repeatable battery of neuropsychological tests: normative values allow application in multiple sclerosis clinical practice. Mult Scler.

[35] Sepulcre J, Vanotti S, Hernández R, Sandoval G, Cáceres F, Garcea O, Villoslada P (2006). Cognitive impairment in patients with multiple sclerosis using the Brief Repeatable Battery-Neuropsychology test. Mult Scler J.

[36] Conners CK (2000). Conners’ Continuous Performance Test user’s manual.

[37] Wechsler D, Kaufman AS, Lichtenberger EO (2001). Wechsler Adult Intelligence Scale III.

[38] Benedet MJ, Alejandre MA (1998). Test de Aprendizaje Verbal España-Complutense (TAVEC).

[39] Buriel Y, Gramunt N, Böhm P (2004). Verbal fluency: preliminary normative data in a Spanish sample of young adults (20-49 years of age). Neurologia.

[40] Krikorian R, Bartok J, Gay N (1994). Tower of London procedure: a standard method and developmental data. J Clin Exp Neuropsychol.

[41] Heaton RK (2001). Test de clasificación de tarjetas de Wisconsin.

[42] Peña-Casanova J, Gramunt-Fombuena N, Gich-Fullà J, Peña-Casanova J, Gramunt-Fombuena N, Gich-Fullà J (2004). Test de orientación de líneas de Benton. Test neuropsicológicos.

[43] Lezak MD, Howieson DB, Bigler ED, Tranel D (2012). Neuropsychological Assessment.

[44] Scherer P (2007). Cognitive screening in multiple sclerosis. J Neurol.

[45] Deeks JJ (2001). Systematic reviews in health care: systematic reviews of evaluations of diagnostic and screening tests. BMJ.

[46] Sampson H (1958). Serial addition as a function of stimulus duration and pacing. Can J Psychol.

[47] Sampson H, MacNeilage PF (1960). Temporal integration and the serial addition paradigm. Aust J Psychol.

[48] Parmenter BA, Weinstock-Guttman B, Garg N, Munschauer F, Benedict RHB (2007). Screening for cognitive impairment in multiple sclerosis using the Dymbol digit Modalities Test. Mult Scler.

[49] Benedict RHB, Munschauer F, Linn R, Miller C, Murphy E, Foley F, Jacobs L (2003). Screening for multiple sclerosis cognitive impairment using a self-administered 15-item questionnaire. Mult Scler.

[50] Benedict RH, Cox D, Thompson LL (2004). Reliable screening for neuropsychological impairment in multiple sclerosis. Multiple Sclerosis Journal.

[51] Benedict RHB, Duquin JA, Jurgensen S, Rudick RA, Feitcher J, Munschauer FE, Panzara MA, Weinstock-Guttman B (2008). Repeated assessment of neuropsychological deficits in multiple sclerosis using the Symbol Digit Modalities Test and the MS Neuropsychological Screening Questionnaire. Mult Scler.

[52] Rudick RA, Cutter G, Reingold S (2002). The multiple sclerosis functional composite: a new clinical outcome measure for multiple sclerosis trials. Mult Scler J.

[53] Sonder JM, Burggraaff J, Knol DL, Polman CH, Uitdehaag BMJ (2014). Comparing long-term results of PASAT and SDMT scores in relation to neuropsychological testing in multiple sclerosis. Mult Scler J.

[54] Rosti E, Hämäläinen P, Koivisto K, Hokkanen L (2007). PASAT in detecting cognitive impairment in relapsing-remitting MS. Applied neuropsychology.

[55] Karabudak R, Dahdaleh MP, Aljumah MA, Alroughani R, Alsharoqi IA, AlTahan AM, Bohlega SA, Daif A, Deleu D, Amous A, Inshasi JS, Rieckmann P, Sahraian MA, Yamout BI (2015). Functional clinical outcomes in multiple sclerosis: current status and future prospects. Mult Scler Relat Disord.

[56] Strober L, Englert E, Munschauer F (2009). Sensitivity of conventional memory tests in multiple sclerosis: comparing the Rao Brief Repeatable Neuropsychological Battery and the Minimal Assessment of Cognitive Function in MS. Mult Scler.

[57] Benedict RH, DeLuca J, Phillips G (2017). Validity of the Symbol Digit Modalities Test as a cognition performance outcome measure for multiple sclerosis. Multiple Sclerosis Journal.

[58] Benedict RH, Fischer JS, Archibald CJ (2002). Minimal neuropsychological assessment of MS patients: a consensus approach. Clin Neuropsychol.

[59] Rao SM (1990). The Cognitive Function Study Group of the National Multiple Sclerosis Society. A manual for the brief repeatable battery of neuropsychological tests in multiple sclerosis.

[60] Houtchens MK, Benedict RHB, Killiany R, Sharma J, Jaisani Z, Singh B, Weinstock-Guttman B, Guttmann CRG, Bakshi R (2007). Thalamic atrophy and cognition in multiple sclerosis. Neurology.

[61] Fink F, Eling P, Rischkau E, Beyer N, Tomandl B, Klein J, Hildebrandt H (2010). The association between California Verbal Learning Test performance and fibre impairment in multiple sclerosis: evidence from diffusion tensor imaging. Mult Scler J.

[62] Ramasamy DP, Benedict RH, Cox JL (2009). Extent of cerebellum, subcortical and cortical atrophy in patients with MS: a case-control study. J Neurol Sci.

[63] Gauthier S, Reisberg B, Zaudig M, Petersen RC, Ritchie K, Broich K, Belleville S, Brodaty H, Bennett D, Chertkow H, Cummings JL, de Leon M, Feldman H, Ganguli M, Hampel H, Scheltens P, Tierney MC, Whitehouse P, Winblad B (2006). Mild cognitive impairment. Lancet.

[64] Huijbregts SCJ, Kalkers NF, de Sonneville LMJ, de Groot V, Polman CH (2006). Cognitive impairment and decline in different MS subtypes. J Neurol Sci.

